# d-Amino Acids Are Exuded by *Arabidopsis thaliana* Roots to the Rhizosphere

**DOI:** 10.3390/ijms19041109

**Published:** 2018-04-07

**Authors:** Claudia Hener, Sabine Hummel, Juan Suarez, Mark Stahl, Üner Kolukisaoglu

**Affiliations:** Center for Plant Molecular Biology (ZMBP), University of Tübingen, Auf der Morgenstelle 32, 72076 Tübingen, Germany; claudia.hener@zmbp.uni-tuebingen.de (C.H.); sabine.hummel@zmbp.uni-tuebingen.de (S.H.); juan.suarez@zmbp.uni-tuebingen.de (J.S.)

**Keywords:** d-amino acids, chiral LC-MS, root exudation, plant-rhizosphere interactions, molecular transport

## Abstract

Proteinogenic l-amino acids (l-AAs) are essential in all kingdoms as building blocks of proteins. Their d-enantiomers are also known to fulfill important functions in microbes, fungi, and animals, but information about these molecules in plants is still sparse. Previously, it was shown that d-amino acids (d-AAs) are taken up and utilized by plants, but their ways to reduce excessive amounts of them still remained unclear. Analyses of plant d-AA content after d-Ala and d-Glu feeding opened the question if exudation of d-AAs into the rhizosphere takes place and plays a role in the reduction of d-AA content in plants. The exudation of d-Ala and d-Glu could be confirmed by amino acid analyses of growth media from plants treated with these d-AAs. Further tests revealed that other d-AAs were also secreted. Nevertheless, treatments with d-Ala and d-Glu showed that plants are still able to reduce their contents within the plant without exudation. Further exudation experiments with transport inhibitors revealed that d-AA root exudation is rather passive and comparable to the secretion of l-AAs. Altogether, these observations argued against a dominant role of exudation in the regulation of plant d-AA content, but may influence the composition of the rhizosphere.

## 1. Introduction

The proteinogenic l-amino acids (l-AAs) are, according to textbook knowledge, ubiquitously found in all living organisms. Many of their functions are essential, especially as primary metabolites and building blocks of proteins. Their enantiomers, the d-amino acids (d-AAs), are also widely distributed in nature, but their functions are still cryptic in many cases. The most prominent example of d-AA utilization is found in bacteria which incorporate d-Ala and d-Glu into their cell wall as structural elements and to protect it from proteases [1]. However, d-AAs are also widespread in eukaryotes: in bound forms within bioactive peptides from crustaceans to vertebrates or within long living proteins from humans (for a review, see [2]). Furthermore, d-AAs also fulfill physiological functions in their free form. A prominent example for such a case is given by the *N*-methyl-d-aspartate (NMDA) receptor in mammals, which binds d-Ser as a co-agonist. In humans, it was found that reduced levels of d-Ser and resulting hypofunction of NMDA receptors leads to schizophrenia [3].

These examples show that the availability of particular d-AAs can be essential for many organisms. In many cases, these organisms are able to produce them de novo, such as in the case of bacteria which possess various types of amino acid racemases. These enzymes, which catalyze the interconversion of l- and d-AAs, are the major drivers of d-AA production in bacteria, but also in animals (for overviews, see [4,5,6]). Another major pool of d-AAs, especially for higher eukaryotes, is their uptake either by nutrition, in animals, or by root uptake, in plants [7,8]. All these organisms are not just dependent on possessing sufficient amounts of particular d-AAs, but also on regulated ways to metabolize them for the prevention of toxic effects by overdosage of particular d-AAs, observable in mammals [7] or plants [9]. Therefore, a major way to catabolize d-AAs is deamination, mostly in an oxidative way by d-AA oxidases (for reviews, see [10,11,12]).

When it comes to d-AA production, uptake, function, and metabolism in plants, there is a remarkable lack of knowledge in these fields compared to other organismal groups. For a long time, d-AAs were regarded as physiologically useless for plants due to their toxicity and low metabolization capacity [13]. However, the physiological value of d-AAs for plants has to be reviewed in the light of recent findings: it has been shown before that not all d-AAs are detrimental to plant growth; even some l-AAs show more inhibitory capacity than their d-enantiomers [8,9]. Furthermore, it has been shown that wheat plants are able to utilize d-Ala as a nitrogen source [14]. However, the functions of d-AAs in plants are not confined to nitrogen delivery. Previously, it has been shown that d-Ser is involved in pollen tube growth in *Arabidopsis* [15]. d-Ala acts as a stress signal in duckweed [16], and it is incorporated into moss chloroplast envelopes as a structural element [17]. Together with the mentioned role of d-Ala as a nitrogen source, d-AAs seem to fulfill a broad range of physiological functions in plants, and many of them remain yet to be unraveled.

In regard to the different functions of d-AAs in plants, their metabolism has come into the focus of plant physiologists. Plant roots are surrounded by d-AAs in their rhizosphere, which are mainly from bacterial origin and are also utilized by bacteria [18,19]. Therefore, it is not astonishing that plants are also able to take up a large variety of d-AAs [20]. With the amino acid transporters LHT1 and ProT2, there are at least two candidates for which d-AA import could be identified [21,22]. Additionally, the ability of plants to synthesize d-AAs de novo has been reported before [23], and with the serine racemase from *Arabidopsis*, also the first d-AA-synthesizing enzyme could be identified in plants [24]. In contrast to the uptake and synthesis of d-AAs, the situation is less clear with respect to the regulation of d-AA content in plants. It has been observed before that exogenously applied d-AAs are partially converted to their l-enantiomers, but all of them are transformed into d-Ala and d-Glu in *Arabidopsis* [8,20]. Recently, it could be shown that a d-amino acid specific transaminase, AtDAT1, is responsible for these processes [25]. However, the question remained about the further fate of d-Ala and d-Glu as major products of this enzyme reaction in plants.

This question takes the center stage of the present study. As one possibility for reducing the d-Ala and d-Glu contents in plants, rhizodeposition has been suggested [26]. In this study, it is shown that exogenously applied d-Ala and d-Glu is significantly reduced in *Arabidopsis* seedlings within 24 h. Furthermore, exudation of these and other d-AAs could be observed. Experiments with uncoupling agents such as CCCP and orthovanadate indicated that the exudation of d- and l-AAs may be a passive mechanism. Although root exudation of d-AAs does not contribute significantly to the reduction of its content in plants, the question about its functions remains and will be discussed.

## 2. Results

### 2.1. d-Ala and d-Glu Are Degraded Rapidly in Seedlings

**Figure 1:**
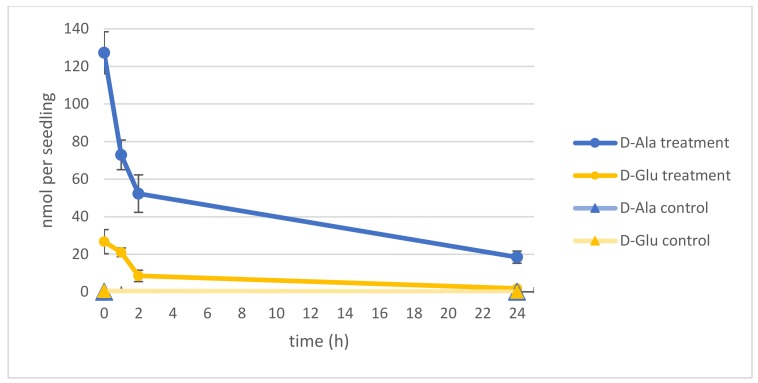
Decrease of d-Ala and d-Glu levels in *Arabidopsis* seedlings within 24 h. Dark blue lines represent the d-Ala and dark yellow lines the d-Glu contents of seedlings, respectively. Measurements from d-amino acid (AA)-treated plants are marked with circles; untreated control plants with lighter colors and triangles. The measurements started directly at transfer to fresh media up to 24 h later. Error bars: ± SD.

In the beginning of our studies was the question about the fate of the major intermediates of d-AA conversion: d-Ala and d-Glu [8,20]. Especially the reduction of the d-Ala content was of interest, due to its relatively high toxicity [8,9]. To analyze the capacity of *Arabidopsis thaliana* seedlings to reduce their d-Ala and d-Glu contents, they were germinated first for 14 d in a liquid medium in 96-well microtiter plates. Then, 1 mM of either d-Ala or d-Glu were applied to the media. After 24 h, seedlings were washed and transferred to fresh media, and seedlings were sampled for another 24 h to analyze their different AA contents. As can be seen in Figure 1, the contents of both d-Ala and d-Glu decreased in the seedlings in this time without reaching the levels of untreated control plants.

**Figure 2:**
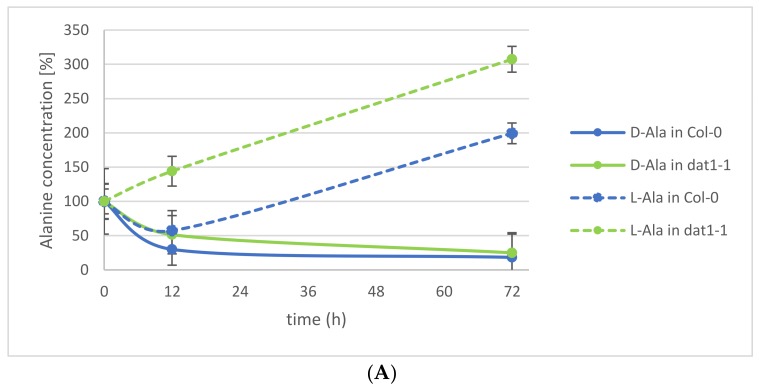

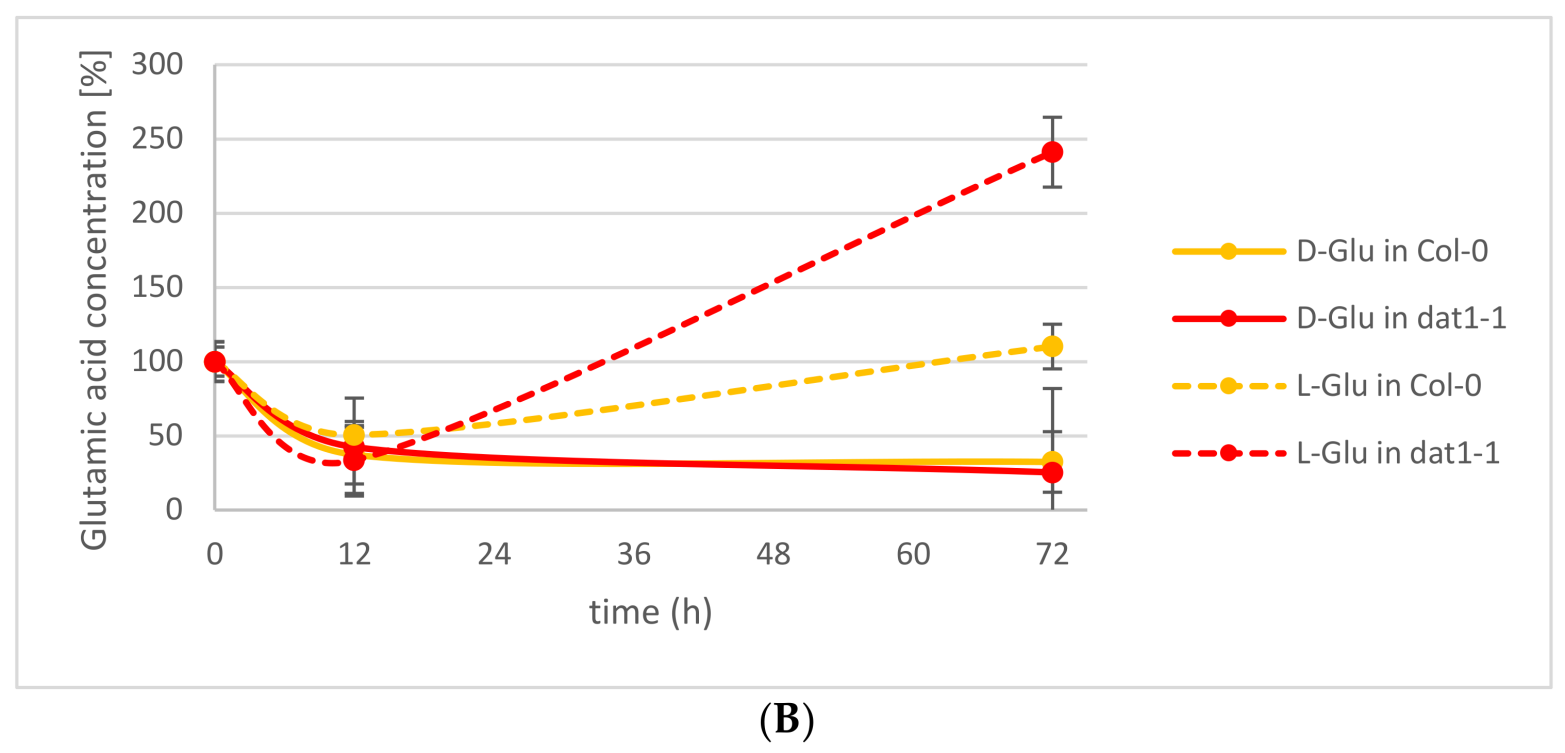
Relative alanine and glutamic acid concentrations in Col-0 wild-type and dat1 mutant line (*dat1-1*) seedlings within 72 h. Seedlings were exogenously applied with (**A**) d-Ala and (**B**) d-Glu, and their d- and l-Ala or d- and l-Glu contents were determined, respectively. Solid lines mark the d-enantiomer; dotted lines the l-enantiomer. In (**A**), blue lines represent Ala from Col-0; green ones Ala from *dat1-1*. In (**B**), yellow lines represent Glu from Col-0; red ones Glu from *dat1-1*. Error bars: ± SD.

This observation led to the question of which processes may contribute to this d-AA reduction in the plants. Various enzymatic and nonenzymatic processes have been suggested elsewhere [26]. Among the putative enzymatic metabolizations of d-AAs, we tested the impact of d-AA-specific transamination. Recently, the responsible enzyme for the almost-complete d-AA transamination activity in *Arabidopsis* could be identified as AtDAT1 [25], an enzyme which had been characterized as a d-Asp transaminase before [27]. The loss of this enzyme leads to the inability of the plants to convert any d-AA into d-Ala and d-Glu, as it has been observed for the *Arabidopsis* accession Landsberg erecta (Ler) [20,25]. To analyze the portion of transamination in the reduction of d-Ala and d-Glu in seedlings, *Arabidopsis* Columbia-0 (Col-0) wild-type and a dat1 mutant line (*dat1-1*) were treated with both d-AAs as described above, and their Ala and Glu contents were determined for 72 h. These analyses revealed that the reduction of d-Ala and d-Glu is not different between both tested lines over the observed time. Instead, the content of the corresponding l-enantiomer of the applied d-AA increases significantly (Figure 2A,B). This implies the involvement of racemases in the reduction of d-Ala and d-Glu levels.

### 2.2. d-AAs Are Exuded by Roots into the Medium

**Figure 3:**
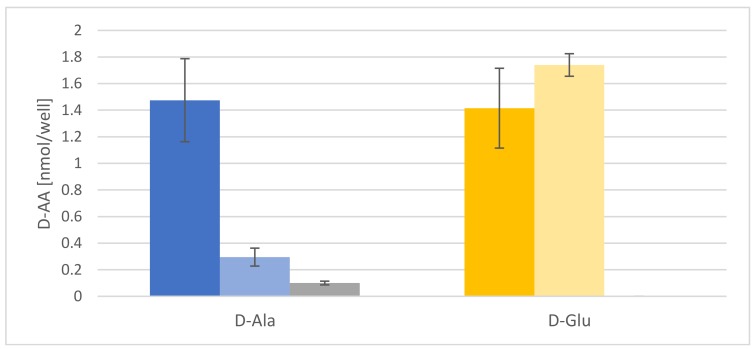
Contents of d-Ala and d-Glu in media released by *Arabidopsis* seedlings treated with both d-AAs. Dark blue and light blue bars represent d-Ala content per well 12 and 72 h after transfer from the d-Ala application medium to fresh medium, respectively. The grey bar represents the 72 h values for media from control plants without d-Ala treatment. Dark-yellow and light-yellow bars represent d-Glu concentration per well 12 and 72 h after transfer from the d-Glu application medium to fresh medium, respectively. d-Glu was not detected in the medium of untreated plants. Error bars: ± SD.

Another possible mechanism for d-AA reduction to be tested was rhizodeposition [26]. It is a well-established fact that plants release proteinogenic AAs into their rhizosphere [28], but the root exudation of d-AAs has not been reported before. Therefore, *Arabidopsis* seedlings were fed with d-Ala and d-Glu, washed, and then transferred to fresh media. These media were then analyzed after 12 and 72 h. The results of these experiments are presented in Figure 3. In both cases, there is a comparable release of both d-AAs to the medium, which is significantly higher than in the untreated control samples. It is noteworthy that the d-Ala concentration in the medium decreases significantly after 72 h, whereas d-Glu stays almost constant.

### 2.3. The Energetization of d-Ala Exudation

**Figure 4:**
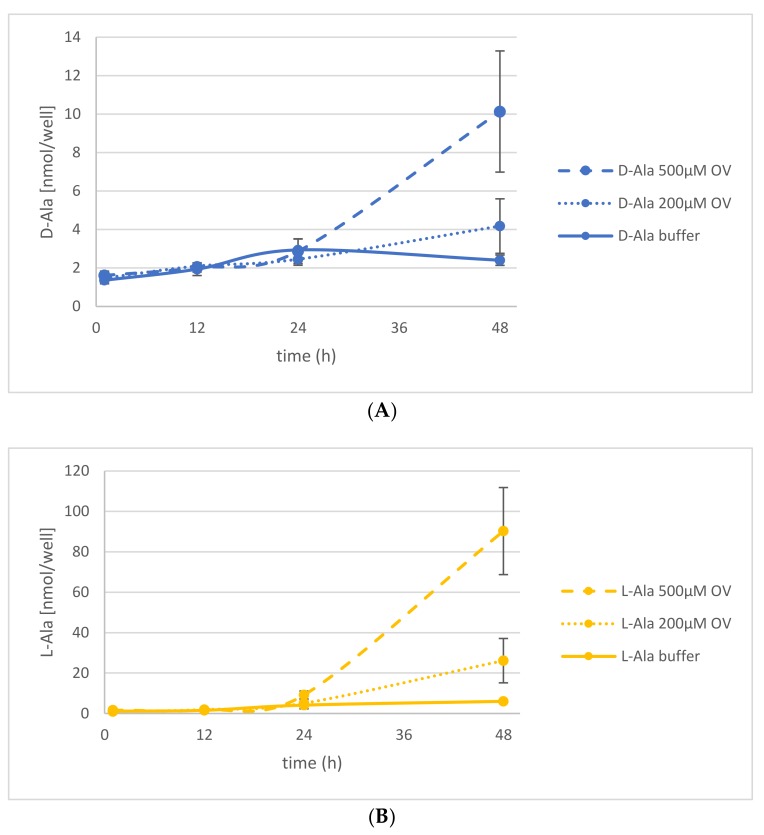
Release of d- and l-Ala into the medium under Na-orthovanadate (OV) treatment. Seedlings were treated with d-Ala and then transferred to fresh medium without OV (solid line), with 200 µM OV (dotted line), and with 500 µM OV (dashed line). Then, the d-Ala (**A**) and l-Ala (**B**) contents in the media were analyzed from 1 to 48 h after transfer. Error bars: ± SD.

The observation of d-AA exudation into the rhizosphere led to the questions of if this process is comparable to the exudation of l-AAs and how it is energized. It has been shown before that l-AA exudation is related to ATP hydrolysis by ATP binding cassette (ABC) transporters [29,30]. To find out if this also holds true for d-AA exudation, seedlings were treated with d-Ala and afterwards transferred to fresh media with Na-orthovanadate (OV) or carbonyl cyanide 3-chlorophenylhydrazone (CCCP). The analysis of the AA composition in the medium after treatment with 200 and 500 µM OV, an inhibitor of ATP hydrolysis, revealed an increase of d-Ala exudation over time compared to the untreated control plants (Figure 4A). In the same time, the d-Ala content within treated and untreated plants decreased comparably (Figure S1A). The release of the corresponding l-enantiomer into the medium also increased upon OV application even to a greater extent (Figure 4B). In contrast to d-Ala, the l-Ala concentration in the plants rose with increasing OV concentration (Figure S1B).

**Figure 5:**
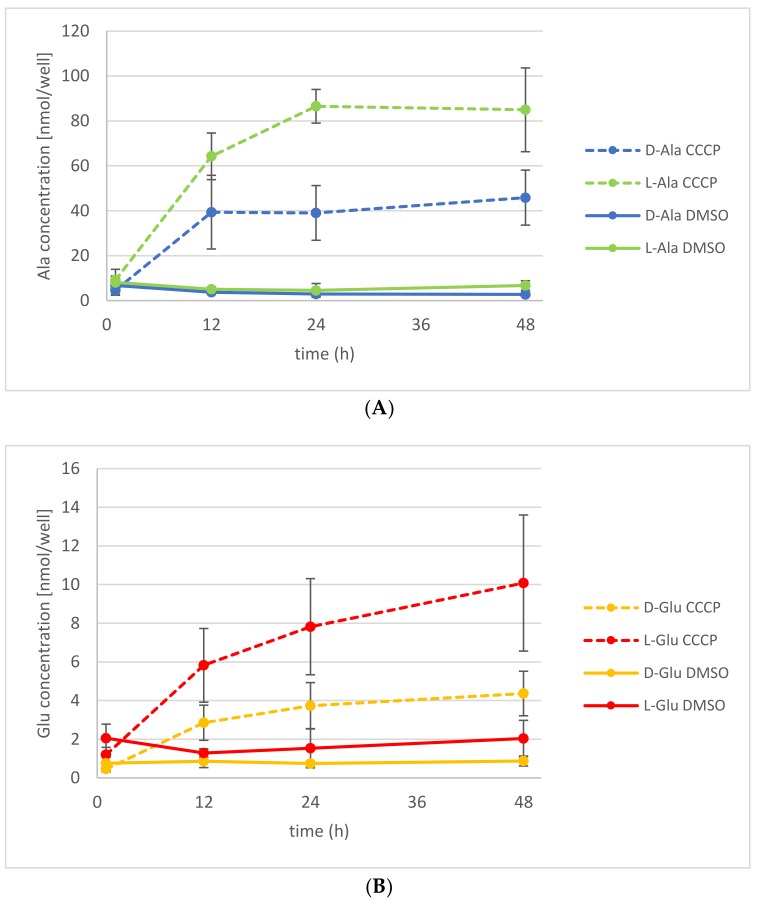
Release of d-/l-Ala and d-/l-Glu into the medium under CCCP treatment. Seedlings were treated with d-Ala and then transferred to fresh medium with 50 µM CCCP (dashed line) or with the solvent of CCCP, dimethyl sulfoxide (DMSO; solid line). (**A**) The d-Ala (blue lines) and l-Ala (green lines) contents in the media were analyzed from 1 to 48 h after transfer. (**B**) In the same media, the d-Glu (yellow lines) and l-Glu (red lines) contents were also determined. Error bars: ± SD.

Application of another ABC transporter blocking agent, CCCP, which causes the dissipation of proton gradients, also led to an increase of d-Ala and l-Ala exudation (Figure 5A). As observed before with OV application, the d-Ala content in the plants decreased too, whereas the l-Ala remained almost constant (Figure S2A). Additionally, it has been shown before that application of d-Ala leads to the accumulation of d-Glu in the plant [20]. Thus, the exudation of d- and l-Glu could also be analyzed without external application. There was an increased exudation of d-Glu observable, as well as of its l-enantiomer, after the application of d-Ala (Figure 5B). There were no significant changes observed of the Glu levels in treated and untreated plants (Figure S2B).

### 2.4. Root Exudation of Other d-AAs

After initial characterization of d-Ala and d-Glu exudation, the question arose if also other d-AAs are secreted in this way. Therefore, five additional d-AAs (d-Asp, d-Leu, d-Lys, d-Phe, and d-Pro) were chosen for seedling treatment and AA release measurement into the medium, as performed before (see Section 2.2, Figure 3). The results of these experiments are summarized in Table 1.

There are some tendencies about AA exudation in this data set attracting interest: First of all, exudation of all additionally tested d-AAs could be detected. The exudation levels for d-Asp and d-Leu were similar to that of their corresponding l-enantiomers, in the beginning. Additionally, the levels of some d-AAs in the medium of d-AA-treated plants decreased over time (d-Asp, d-Leu, d-Lys, and d-Phe), whereas d-Pro levels in the medium stayed constant over time. Both exudation patterns (decreasing and constant ones) were also observed for d-Ala and d-Glu, respectively (Figure 3, Figure 4 and Figure 5).

## 3. Discussion

In the beginning of this study, there was the question about the fate of d-Ala and d-Glu in plants, being the major conversion products of d-AA metabolism. The presented analyses revealed that exogenously applied d-Ala and d-Glu were reduced in the plants to less than one-fifth within the first 24 h (Figure 1), and further reduction of the remainder needed more than 72 h (Figure 2A,B). Although these results do not answer the initial question finally, they point to different mechanisms involved in d-Ala and d-Glu reduction in plants. The decrease of d-Ala and d-Glu contents and the simultaneous increase of their respective l-enantiomers after d-AA feeding (Figure 2A,B) imply an enzymatic interconversion of d- to l-AA. The most obvious reaction to explain this interconversion would be racemization. Although biochemical evidences for a plant alanine racemase have been found previously in *Chlamydomonas* [31] and *Medicago* [32], the identification of its encoding gene in plants is still pending. Additionally, an enzyme in plants with glutamate racemase activity has not yet been reported.

However, there are also indirect ways to degrade d-Ala and d-Glu or to form l-Ala and l-Glu from them. The easiest one in this respect would be the deamination of the d-AAs by an oxidase, lyase, or dehydrogenase to NH_3_ and its corresponding keto acids. The subsequent transamination of the keto acid by an l-AA transaminase would then result in the formation of the corresponding l-AAs. However, the reports about d-AA-deaminating enzymes in plants, especially d-AA oxidases, are scarce and contradictory. Although it has been stated before that plants possess low capacity to metabolize d-AAs and lack d-AA oxidases [13], there is also a report biochemically characterizing a putative d-AA oxidase from corn [33]. Furthermore, the *Arabidopsis* genome harbors at least one putative d-AA oxidase gene [26].

Another possibility of d-Ala and d-Glu reduction in our experiments is d-AA-specific transamination, as implied in previous studies. Previously, it was found that almost all d-AAs are converted into d-Ala and d-Glu; also both d-AAs into each other [8]. In a follow-up report [20], the *Arabidopsis* accession *Ler* was found to be unable to perform this conversion. Later, it was shown that a defective DAT1 protein in this accession is responsible for this effect, which leads to the loss of this major determinant in plant d-AA metabolism [25]. In Figure 2A,B, it can be seen that reduction of the tested d-AAs does not differ between the wild-type and the *dat1* mutant. This shows, at least, that plants possess alternatives to DAT1-catalyzed transamination with comparable capacity to metabolize d-AAs, which await to be unraveled.

Another principal way of reducing the contents of d-Ala and d-Glu in plants would have been exudation, which was another major focus of this study. The presented data revealed that d-Ala and d-Glu, as well as all other tested d-AAs, were secreted to the rhizosphere in amounts of <10 nmol per seedling (Table 1, Figure 5A,B). Despite the fact that the levels of d-Ala and d-Glu within the plants were about an order of magnitude higher, the d-AA levels in the medium did not increase over time significantly (Figure 4 and Figure S1, Figure 5 and Figure S2). Therefore, the exudation of d-AAs does not seem to contribute crucially to the reduction of these compounds in plants. Instead, the d-Ala and d-Glu levels remain constant in the media over time, but increase drastically after inhibition of active transport (Figure 4 and Figure 5).

This leads to the question of how this observation can be explained. It is a long-lasting matter of debate whether root secretion is a passive or actively energized process (for summaries, see [28,34]). In respect to the exudation of l-AAs, there are two publications reporting aberrant l-AA profiles in ATP-binding cassette (ABC) transporter mutant lines: In one case, the knockout (KO) of AtMRP2, belonging to the multidrug resistance-related protein (MRP) subclass of ABC transporters, leads to a significant increase of l-Pro, l-Tyr, l-Phe, and l-Ala in root exudate [28]. In the other case, the loss of AtMRP5 causes the opposite effect, where all analyzed l-AAs are decreased in the exudate [30]. In our inhibitor experiments in the present report, the secretion of d-Ala and d-Glu was increased by OV, a general ABC transport inhibitor [35], and CCCP, a potent protonophore and inhibitor of metabolically active processes [36]. Furthermore, the content of the corresponding l-enantiomers was also higher in exudates of inhibitor-treated seedlings (Figure 4 and Figure 5).

These observations point to two things: First, the exudations of d- and l-AAs, as far as it concerns Ala and Glu, are similarly regulated processes. This is interesting insofar that knowledge from previous studies about l-AA exudation [36] may also be transferred to root secretion of their d-forms. However, this has to be confirmed in future studies. Furthermore, it would be worthwhile for the future to analyze the chirality of AAs in exudates, as the presented results showed that l- and d-forms are both secreted (Figure 5 and Figure S2). Second, there is at least one passive efflux process, which contributes to AA exudation to a certain extent. If uptake and exudation are exclusively active processes, one would expect either a block of exudation after OV and CCCP treatment or at least a decrease by increasing or prolonged inhibitor treatment. However, the opposite takes place (Figure 4 and Figure 5). For the moment, the best explanation for this scenario would be that a crucial portion of exudation runs via passive efflux, whereas a significant part of the uptake, especially the reuptake of secreted AAs, is ATP-dependent. This scenario is supported by several findings: The uptake of d-Ala in *Arabidopsis* is primarily achieved by the AA transporter LHT1 [8,21], which is CCCP-sensitive [37]. Reuptake of d-Ala and other d-AAs has been observed before [25]. Also, AA root secretion was assumed before not to be energized [28,34,38].

Although the suggested scenario is a working hypothesis and needs to be thoroughly confirmed, it can be concluded that d-AAs are part of the composition of plant root exudates. This finding leaves and opens several questions. For instance, it is still open if just the tested d-AAs, or even all of them, can be secreted, as the results in Table 1 imply. In this respect, it would also be interesting as to what extent the d-AA concentration in the plant influences the exudation process and rate. Together with the transport aspects of the d-AAs, the responsible transporters need to be identified. Several l-AA transporter families in plants have been identified and characterized in the past (for a latest review, see [39]). For some of their members, even the transport of d-AAs was shown: besides the role of AtLHT1 in d-Ala uptake (see above), AtProT1 and AtProT2 have been shown to transport d-Pro [22], and AtAAP1 seems to be involved in d-Met uptake [26]. These examples show that the known amino acid transporters are also able to facilitate d-AA transport and maybe also contribute to their root exudation.

All the questions mentioned before lead to the physiological role of d-AA root exudation. Generally, root exudates are composed of a large variety of chemical compounds, and have been once classified either as mediating plant–plant interactions or playing a role in plant–microbe interactions [40]. In respect to the latter interactions, the exudates may fulfill a rhizosphere-forming function by defending the plant from pathogenic microbes. A possible role of the exudation of d-AAs may be also the attraction of specific microorganisms [28,34]. It has been shown before that the amino acid content of root exudates influences the soil either towards production of antibacterial or antifungal volatiles [41]. Additionally, d-AAs are also generally utilized by bacteria and are able to grow on them as a sole source of carbon and nitrogen [19,42,43]. These properties make d-AAs a potential attractant for bacteria. When it comes to the impact of d-AAs on plant-plant interactions, a possible role of them may be neighbor recognition. The influence of root exudates on kin recognition has been discussed before [44]. Furthermore, nonproteinogenic amino acids, such as meta-tyrosine, produced by plants can act as herbicides and inhibit the growth and settling of other species [45]. Future studies will show if d-AA exudation has an impact on rhizosphere composition, but also on the growth and composition of plant populations.

## 4. Materials and Methods 

### 4.1. Chemicals

MS media components were purchased from Duchefa (Haarlem, The Netherlands). To apply, determine, and quantify amino acids in plant extracts and media, standard materials were purchased from Sigma-Aldrich (Steinheim, Germany) or in LC/MS grade from Roth (Karlsruhe, Germany).

### 4.2. Plant Material and Growth Conditions

*Arabidopsis* seeds of the *dat1-1* mutant and its corresponding wild-type Col-0 were ordered from the Nottingham *Arabidopsis* Stock Centre (University of Nottingham, UK) and genetically characterized as described elsewhere [23]. All seedlings were germinated for 14 d under long-day conditions in microtiter plates as described before [18]. For d-AA uptake and metabolization analyses, d-AAs were added to the media to a final concentration of 1 mM for 24 h. Subsequent transfer into fresh media was done after two washing steps in MilliQ water and brief drying of the seedlings on tissues to remove excess water. Growth conditions were the same as given above.

### 4.3. Extraction of Amino Acids from Seedlings and Media and Their Derivatization

Amino acid extraction from seedlings and derivatization of AAs in solution were performed as described elsewhere [8]. To derivatize AAs in media, the buffer conditions were adjusted to 0.1 M Tris/HCl, pH 8, such as in plant extracts, by adding 1 M Tris/HCl. The incubation time of derivatization was elongated to 3 h and the derivatized liquid volume was adjusted with acetonitrile instead of methanol for all derivatizations.

### 4.4. LC/MS Determination of d- and l-AAs

In the course of the studies, the following amino acids were measured: d/l-alanine, d/l-aspartate, d/l-glutamate, d/l-leucine, d/l-lysine, d/l-phenylalanine, and d/l-proline. An Acquity–SynaptG2 LC/MS system from Waters (Manchester, UK) was used for quantification, and operated in positive electrospray ionization mode. The mass spectrometer was operated at a capillary voltage of 3000 V and a resolution of 20,000. Separation of the abovementioned amino acids was carried out on a RP Acquity HSST3 1 × 150 mm, 1.8 µm column with a flow rate of 50 µL/min and a 22 min gradient from 70% water to 99% acetonitrile (both with 0.1% formic acid). For quantification, 3 µL of sample were injected with a 6-point calibration from 0.125 µM to 1250 µM.

## Figures and Tables

**Table 1:** d- and l-AA contents of media (in nmol/well) 12 and 72 h after 24 h treatment in different d-AAs and transfer to fresh media.

		d-AA Treated Plants				Control Plants			
		d		l		d		l	
		mean	(±SD)	mean	(±SD)	mean	(±SD)	mean	(±SD)
12 h	Aspartate	1.38	(±0.30)	2.15	(±0.35)	0.01	(±0.01)	1.85	(±0.39)
	Leucine	0.32	(±0.03)	0.28	(±0.21)	0.01	(±0.01)	0.14	(±0.13)
	Lysine	1.46	(±1.03)	0.03	(±0.02)	0.00	(±0.00)	0.00	(±0.00)
	Phenylalanine	0.46	(±0.10)	0.00	(±0.00)	0.00	(±0.00)	0.00	(±0.00)
	Proline	2.44	(±0.62)	0.18	(±0.02)	0.76	(±0.16)	0.29	(±0.06)
72 h	Aspartate	0.77	(±0.08)	5.54	(±1.51)	0.03	(±0.01)	5.90	(±0.53)
	Leucine	0.12	(±0.09)	0.00	(±0.00)	0.00	(±0.00)	0.00	(±0.00)
	Lysine	0.20	(±0.16)	0.00	(±0.00)	0.00	(±0.00)	0.00	(±0.00)
	Phenylalanine	0.06	(±0.06)	0.00	(±0.00)	0.00	(±0.00)	0.00	(±0.00)
	Proline	2.48	(±0.27)	0.01	(±0.01)	1.27	(±0.09)	0.09	(±0.06)

## Supplementary Materials

Supplementary materials can be found at http://www.mdpi.com/1422-0067/19/4/1109/s1.
