# Supplementary material for: d-Amino Acids Are Exuded by Arabidopsis thaliana Roots to the Rhizosphere
Source: Int J Mol Sci. 2018 Apr 7;19(4):1109. doi: 10.3390/ijms19041109 (PMC5979410; doi:10.3390/ijms19041109)
Supplement: Supplementary file 1 [file ijms-19-01109-s001.docx]

Supplementary Materials： D-Amino Acids are Exudated by *Arabidopsis Thaliana* Roots to the Rhizosphere

Claudia Hener, Sabine Hummel, Juan Suarez, Mark Stahl and Üner Kolukisaoglu

| (**A**) |
| --- |
| (**B**) |

**Figure S1.** Contents of D- and L-Ala in seedlings under OV treatment. Seedlings were treated with D-Ala and then transferred to fresh medium without (solid line), with 200 µM OV (dotted line) and 500 µM OV (dashed line). Then the D-Ala (A) and L-Ala (B) contents in the seedlings were analyzed from 1-48 h after transfer. Error bars: ±SD.

| (**A**) |
| --- |
| (**B**) |

**Figure S2.** Contents of D-/L-Ala and D-/L-Glu in seedlings under CCCP treatment. Seedlings were treated with D-Ala and then transferred to fresh medium with DMSO (solid line) or with 10 µM CCCP (dotted line). (A) The D-Ala (blue lines) and L-Ala (green lines) contents in the seedlings were analyzed from 1-48 h after transfer. (B) In the same media also the D-Glu (yellow lines) and L-Glu (red lines) contents of the seedlings were determined. Error bars: ±SD.
